# Supplementary material for: (−)-Adaline from the Adalia Genus of Ladybirds Is a Potent Antagonist of Insect and Specific Mammalian Nicotinic Acetylcholine Receptors
Source: Molecules. 2022 Oct 20;27(20):7074. doi: 10.3390/molecules27207074 (PMC9611057; doi:10.3390/molecules27207074)

molecules

*Supplementary Material for*

# (−)-Adaline from the *Adalia* genus of ladybirds is a potent antagonist of insect and specific mammalian nicotinic acetylcholine receptors

**David P. Richards[1], Rohit N. Patel[1], Ian R. Duce[1], Bhupinder P.S. Khambay[2], Michael A. Birkett[2], John A. Pickett[2,3] and Ian R. Mellor[1,*]**

1 School of Life Sciences, University of Nottingham, University Park, Nottingham, NG7 2RD, UK.
2 Rothamsted Research, West Common, Harpenden Hertfordshire, AL5 2JQ, UK.
3 School of Chemistry, Cardiff University, Cardiff, CF10 3AT, UK.
* Correspondence: ian.mellor@nottingham.ac.uk; Tel.: +44 1159 513257

## Contents

Figure S1 ........................................................................ 2

**List of abbreviations used:** ACh: acetylcholine; nAChR: nicotinic acetylcholine receptor; DCM: dichloromethane; DMEM: Dulbecco's modified Eagle medium; EGTA: ethylene glycol-bis(β-aminoethyl ether)-*N,N,N′,N′*-tetraacetic acid; FCS: foetal calf serum; HEPES: *N*-(2-hydroxyethyl)piperazine-*N′*-(2-ethanesulfonic acid)

molecules

MDPI

**Figure S1.** GC-MS analysis. Main graph: gas chromatogram (Agilent 5890 GC, Agilent Technologies, Santa Clara, California, USA) of the *A. bipunctata* alkaloid fraction showing the major peak of (−)-adaline present in the sample. Insert: coupled 70 eV mass spectrum of (−)-adaline.

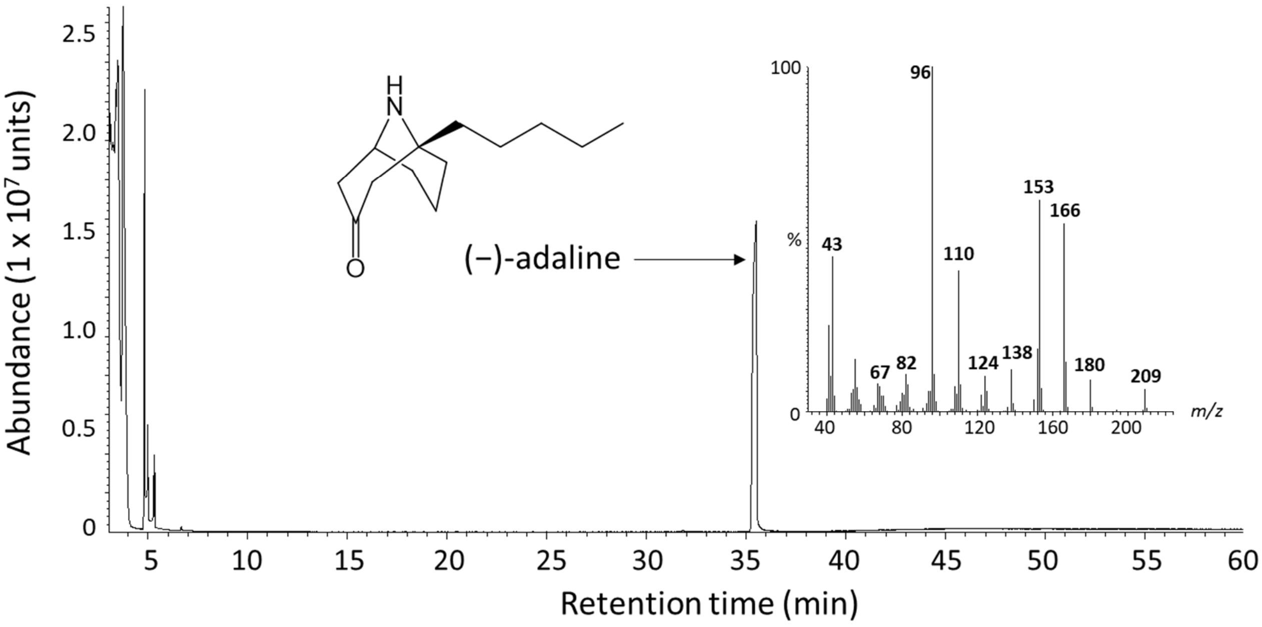

Supplement: Supplementary file 1 [file molecules-27-07074-s001.zip › molecules-1968550-supplementary.pdf]
